# Electroluminescent Liquid Metal Marbles for Reconfigurable Multi‐Color Display

**DOI:** 10.1002/advs.202512263

**Published:** 2025-08-28

**Authors:** Ruohan Yu, Yuan Chi, Richard Fuchs, Shih‐Hao Chiu, Yuanzhu Mao, Shuhua Peng, Priyank Kumar, Kourosh Kalantar‐Zadeh, Jianbo Tang

**Affiliations:** ^1^ School of Chemical Engineering University of New South Wales (UNSW) Kensington NSW 2052 Australia; ^2^ Frontier Science Center for Smart Materials School of Chemical Engineering Dalian University of Technology Dalian 116024 China; ^3^ School of Chemical and Biomolecular Engineering University of Sydney Darlington NSW 2008 Australia; ^4^ Department of Materials Science and Engineering School of Engineering and Research Center for Industries of the Future Westlake University Hangzhou 310030 China; ^5^ School of Mechanical and Manufacturing Engineering University of New South Wales (UNSW) Kensington NSW 2052 Australia

**Keywords:** color display, electric discharge, electroluminescence, liquid metal marbles, liquid metals

## Abstract

Conventional display technologies rely on rigid architectures, limiting their adaptability for reconfigurable systems. Plasma discharge, as a field‐driven excitation method, offers great opportunities for visual interfaces, yet integrating it into controllable and adaptable color display platforms remains challenging. Here, configurable and adaptable electroluminescent platforms based on the plasma discharge of phosphor‐coated liquid metal marbles based on eutectic gallium indium liquid metal droplets are presented. Electroluminescent phosphors emitting the red, green, and blue primary colors are used as a functionalizing coating for the droplets. Mixing different types of phosphor particles at controllable ratios fine tunes the electroluminescent color emitted from individual air gaps between adjacent liquid metal marbles. Such a particle‐mixing‐enabled additive color mixing strategy enables bright color emission across the whole visible spectrum and plasma‐discharge‐based pixelated multicolor display of diverse reconfigurable patterns. This low‐cost and easily reconfigurable liquid metal marble platform offers a multicolor display technique for future displays.

## Graphical abstract

Phosphor‐coated liquid metal marbles offer a reconfigurable electroluminescent multicolor display platform, where the plasma discharge across marble arrays generates colored light. By tuning the mixing ratio of different types of primary‐color‐emitting phosphor particles, full‐spectrum additive color mixing is achieved. Enabling dynamic and programmable multicolor displays, it offers an easy‐to‐fabricate method for novel pixelated display and visualization.

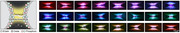

## 1 Introduction

Modern full‐color display technologies predominantly rely on solid‐state light emitting diodes^[^ ^1^ ^]^ and plasma technologies,^[^ ^2^ ^]^ integrated into panel architectures. In this regard, high‐voltage electric discharge, for producing plasma, offers a compelling light‐generation mechanism, characterized by intense visible emission and low current density.^[^ ^3^ ^]^ While these plasma‐based display systems can achieve excellent resolution and efficiency, they generally rely on complex fabrication procedures under highly rigid and structured processes.^[^ ^4^ ^]^ Introducing new versatilities and functionalities into plasma technologies is essential. Whereas these technologies are well‐developed on large industrial scales,^[^ ^2^, ^4^ ^]^ small‐scale and laboratory based processes for customizable and reconfigurable displays remain largely underexplored. There exists a clear gap between the demand for quick, customizable, and easy‐to‐fabricate plasma display platforms and the current technological capabilities. Bridging this gap requires innovative strategies, especially those capable of generating reconfigurable and full‐color optical patterns.

Highly conformable liquid metals can be integrated into plasma displays to cover the discussed gap. Liquid metals are metallic and inherently conductive,^[^ ^5^ ^]^ that provide the needed sites for establishing electrical connections.^[^ ^6^ ^]^ Micro and nanoparticles made of a variety of materials, in particular luminescence particles, can be directly embedded into the surface of liquid metals to achieve versatile, yet stable, surface functionalization.^[^ ^7^ ^]^ Such liquid metal droplets with surface embedded micro and nanoparticles are called “liquid metal marbles”.^[^ ^7b^ ^]^ The stability of these liquid metal marbles is guaranteed by the presence of a few‐nanometer‐thick oxide layer naturally formed on the liquid metal surface in ambient.^[^ ^8^ ^]^ This oxide shell facilitates particle adhesion, allowing the creation of liquid metal marble platforms without the need for complex deposition tools for placing functional layers and surface coatings.

We recently demonstrated an approach using phosphor‐coated liquid metal marbles for optical displays based on the plasma discharge and electroluminescence phenomena.^[^ ^9^ ^]^ In that work, liquid metal marbles coated with copper‐doped zinc sulfide (ZnS:Cu) serve as discrete conductive nodes^[^ ^10^ ^]^ and electroluminescent units for generating localized light emission.^[^ ^9^, ^11^ ^]^ When arranged into patterned marble arrays, a sufficiently high voltage creates individual (pixelated) plasma sparks that discharge across the air gaps between neighboring marbles, producing bright discharge paths. This initial work validated the feasibility of using liquid metal marble arrays as a versatile, easy‐to‐establish, and reconfigurable platform for discharge‐guided optoelectronic displays. However, the essential capabilities for realizing multicolor displays and long‐term stability were not demonstrated.

We here introduce an additive color mixing method based on direct particle mixing to develop liquid metal marble platforms into a multi‐color display. Additive color mixing is widely used in current display technologies,^[^ ^12^ ^]^ where a pixel has three adjacent light‐emitting units generating the red (R), green (G), and blue (B) primary colors independently.^[^ ^13^ ^]^ The color mixing is achieved by generating controlled amounts of the primary colors at each unit, and due to the color averaging effect, it is precepted as a single color defined by the mixing ratio of the primary colors.^[^ ^14^ ^]^ We show that with our liquid metal marble platform, color mixing can be directly achieved by particle mixing. Y_2_O_3_:Eu^3^⁺, ZnS:Cu, and ZnS:Cu,Al phosphors are used as the coating particles for generating the RGB colors, respectively. Mixing the phosphor particles allows the emission color to be tuned, following the additive color mixing rule.^[^ ^12^ ^]^ These color‐emission‐tunable liquid metal marbles are arranged into predesigned patterns to generate highly diverse color displays and we show their performance in different examples.

## 2 Results

### 2.1 Fabrication of Electroluminescent Liquid Metal Marbles

A multi‐color electroluminescent pixelated display platform was developed using liquid metal marbles coated with phosphor micro particles. Room‐temperature liquid metal eutectic gallium–indium (EGaIn) was chosen so that the marbles could remain in liquid state under our experimental conditions.^[^ ^15^ ^]^ Phosphor particles of Y_2_O_3_:Eu^3^⁺, ZnS:Cu, and ZnS:Cu,Al were selected as the electroluminescent coating of the liquid metal droplets. Due to the electroluminescent effect of the phosphor shell, these liquid metal marbles were able to emit luminescent light with the primary colors of red (Y_2_O_3_:Eu^3^⁺),^[^ ^16^ ^]^ green (ZnS:Cu),^[^ ^17^ ^]^ and blue (ZnS:Cu,Al).^[^ ^18^ ^]^

**Figure 1:**
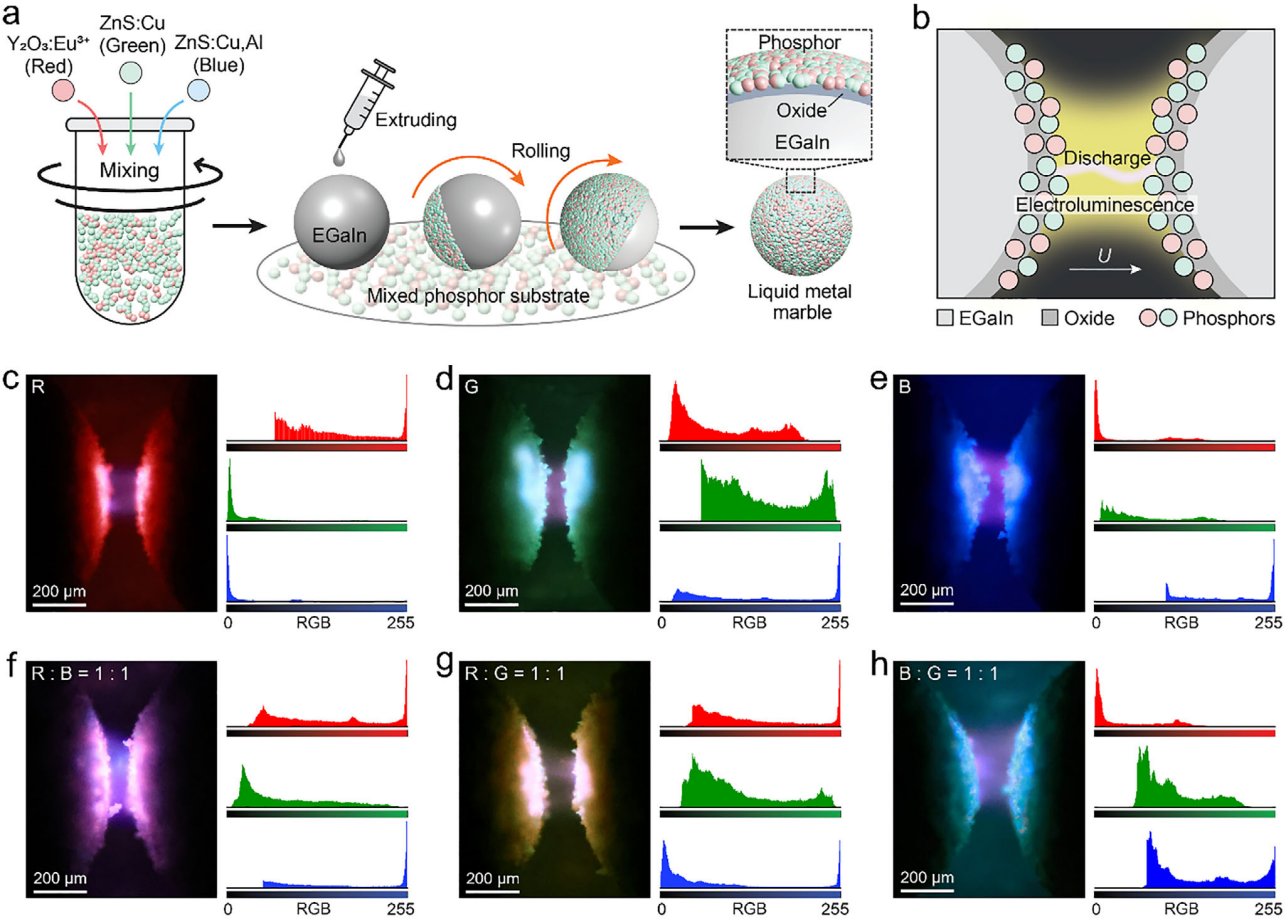
a). Schematic of the fabrication processes and the cross‐section structure of the liquid metal marbles. b) Schematic of the electroluminescence and plasma discharge formed in the air gap between two liquid metal marbles. c–e) Optical images and corresponding RGB intensity profiles for liquid metal marbles coated with a single‐type phosphor particle of: (c) Y_2_O_3_:Eu^3^⁺, (d) ZnS:Cu, and (e) ZnS:Cu,Al. f–h) Optical images and corresponding RGB intensity profiles for liquid metal marbles coated with two types of phosphors at a 1:1 ratio: (f) Y_2_O_3_:Eu^3^⁺ and ZnS:Cu,Al, (g) Y_2_O_3_:Eu^3^⁺ and ZnS:Cu, and (h) ZnS:Cu and ZnS:Cu,Al. The y‐axis in Figures c–h displays the pixel intensity histogram, where values present the frequency count of pixels at each intensity level.

As illustrated in **Figure** **1**a, EGaIn droplets of controlled volume (1.6 µL) were fabricated by an extruding method using a syringe pump. The liquid metal droplets rolled over a phosphor powder bed, during which the phosphor particles adhered to the surface of the EGaIn droplets to form a compact uniform coating. The adhesion between the particles and the liquid metal is facilitated by the surface oxide layer naturally formed on the liquid metal surface, within which the phosphor particles are partially embedded.^[^ ^19^ ^]^ As such, the liquid metal marbles feature a metallic liquid core enclosed by a native gallium oxide shell, which anchors the phosphor particles.

When a sufficiently strong electric field is applied between adjacent liquid metal marbles, the air gaps between the marbles undergo an electrical breakdown, which leads to an electric plasma discharge accompanied by electroluminescent emitting (Figure 1b).^[^ ^9^, ^20^ ^]^ In this study, a compact Tesla coil with adjustable output voltage and frequency was used as the power source. The output voltage was set to 18 kV throughout the experiments and was estimated by fitting the maximum discharge distance into Paschen's law^[^ ^21^ ^]^ (Figure S1, Supporting Information). These electric discharges pass through the liquid metal marbles with a highly conductive liquid metal cores,^[^ ^22^ ^]^ forming transient electroluminescent pathways.^[^ ^23^ ^]^ The electroluminescent color is determined by the light‐emitting behavior of the phosphor particles, and it can be changed by selecting different phosphor materials. As shown in Figure 1c–e, red, green, and blue luminescent light forms between the marbles coated with Y_2_O_3_:Eu^3^⁺, ZnS:Cu, and ZnS:Cu,Al particles, respectively. Their corresponding RGB intensity profiles confirm the spectral dominance of the intrinsic electroluminescent color of the phosphor particles in each case. These color emissions have distinct mechanisms: the red emission of Y_2_O_3_:Eu^3^⁺ arises from the ⁵D_0_ → ⁷F_2_ transition of Eu^3^⁺ ions,^[^ ^16^, ^24^ ^]^ while the green and blue emissions of ZnS:Cu and ZnS:Cu,Al stem from defect‐level transitions involving Cu⁺ centers, with Al^3^⁺ co‐doping shifting the emission toward shorter wavelengths.^[^ ^25^ ^]^ A purple emission due to the plasma discharge of air was seen in all three cases, which has a lower intensity compared to the emission by the phosphor particles. Therefore, these colors are close to but not strictly the primary colors. The origin of the plasma‐induced emission by air electric discharge was confirmed by conducting the experiment in an argon environment. A high‐intensity vibrant purple emission, which is the signature of argon discharge and distinct from discharge in air, was observed (Figure S2, Supporting Information).

Importantly, our strategy allows the electroluminescent color to be fine‐tuned by mixing different types of phosphor particles in predefined ratios. As shown in Figure 1f–h, magenta, yellow, and cyan emissions were obtained by mixing the red‐/blue‐emitting, red‐/green‐emitting, and green‐/blue‐emitting phosphor particles all at a 1/1 ratio. RGB profile analysis of the high‐intensity (high RGB value) regions shows that the electroluminescent emission spectra are superpositions of individual spectra of the phosphor types present in the liquid metal marble coating. These results demonstrate that particle mixing is able to realize additive color mixing using our method.

### 2.2 Characterization of Phosphor‐Coated Liquid Metal Marbles

**Figure 2:**
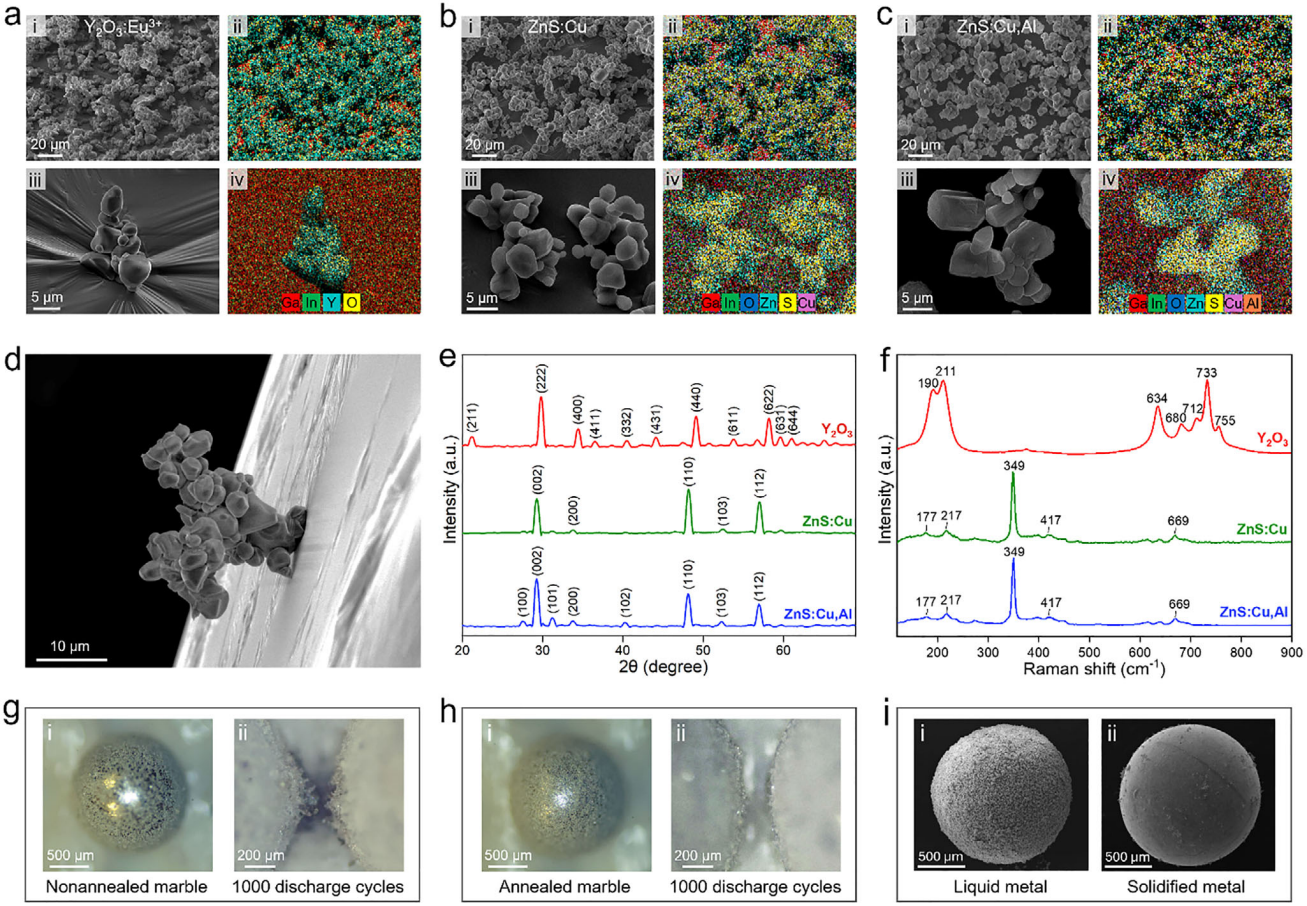
a–c) SEM images and EDS maps of (a) Y_2_O_3_:Eu^3^⁺, (b) ZnS:Cu, and (c) ZnS:Cu, Al phosphor particles coated on the surface of EGaIn droplets: morphologies at different magnifications (i, iii) and element distributions (ii, iv). d) SEM image of a cluster of phosphor particles anchored onto EGaIn surface, showing the strong adhesion between the particles and the liquid metal surface oxide. e) XRD patterns and f) Raman spectra of different types of phosphor particles. g, h) Optical images of (g) nonannealed and (h) annealed marbles before and after 1000 discharge cycles. i) Comparison of particle coating on liquid metal EGaIn (i) and solidified Ga metal (ii).

The phosphor particles coated on the liquid metal marbles were characterized in terms of their composition and morphology. Scanning electron microscopy (SEM) and energy‐dispersive X‐ray spectroscopy (EDS) reveal that different types of micrometer phosphor particles (size range: ≈3–9 µm) and particle clusters form a dense shell on the liquid metal surface (**Figure** **2**a–c). Analyses of representative SEM images show that the surface coverages of the three phosphor types are consistently ≈70% (Figure S3, Supporting Information).

The successful attachment of the particles to the surface of EGaIn marbles is enabled by the native surface oxide layer naturally formed in air. The embedding of the particles into the oxide layer is easily observable through the wrinkled topology of the surface oxide (Figure 2a; Figure S4, Supporting Information). In addition, it is observed that particle clusters are able to be stably anchored to an inclined liquid metal surface at a small anchoring point (Figure 2d), indicating a strong adhesion between the particles and the surface oxide. This oxide‐mediated adhesion ensures the particles to be intact during repeated discharge cycles. X‐ray diffraction (XRD) and Raman characterization confirm the compositional and crystalline structures of the phosphor particles coating on the liquid metal droplets (Figure 2e,f). In addition, no noticeable compositional change takes place during marble fabrication.

While the phosphor particles adhere tightly to the liquid metal surface and the liquid metal marble show good stability under repeated discharge, the discharging spots of the marbles can experience considerable deformation after, for instance, 1000 discharge cycles (Figure 2g). To counteract this deformation, we introduce a thermal annealing step to improve the particle‐oxide adhesion and overall marble stability by increasing the oxide layer thickness during the heating process in air. It was shown that after being annealed at 200 °C for 30 min, the liquid metal marbles experience no noticeable change in appearance after 1000 discharge cycles (Figure 2h). Furthermore, the annealed marbles remain functional when discharged > 15 000 times, after which the change in inter‐marble gap distance and marble shape is minimal (Figure S5, Supporting Information). Such a significant improvement in stability is attributed to the thickening of oxide as well as the strengthened interaction between the particles and the oxide during annealing. A comparison of the Raman spectra of the EGaIn droplets, with no particles coating, before and after annealing, reveals the emergence of a pronounced broad band ≈700 cm^−1^ in the annealed samples. This spectral feature indicates that the oxide skin becomes thicker, accompanied by the dehydration of hydroxylated Ga oxide species to form Ga_x_O_y_ (Figure S6, Supporting Information).^[^ ^26^ ^]^ The improvement of particle adhesion and structural stability of the liquid metal marbles, after annealing, was also validated by subjecting both the annealed and non‐annealed marbles to long‐term gentle rolling and shaking in a petri dish. After such mechanical agitations, the non‐annealed marbles exhibited noticeable surface collapse and particles detachment, while the annealed marbles maintained their overall spherical shapes and particles coverage (Figure S7, Supporting Information).

It should be noted that the liquid conformable state of the marbles is also crucial to realize particle attachment. When solidified gallium spheres (instead of liquid metal droplets) were used, a compact particle coating could not be formed (Figure 2i) as phosphor particles could not embed themselves through the skin into the liquid anymore.

### 2.3 Decay Dynamics of Electroluminescent Emission

**Figure 3:**
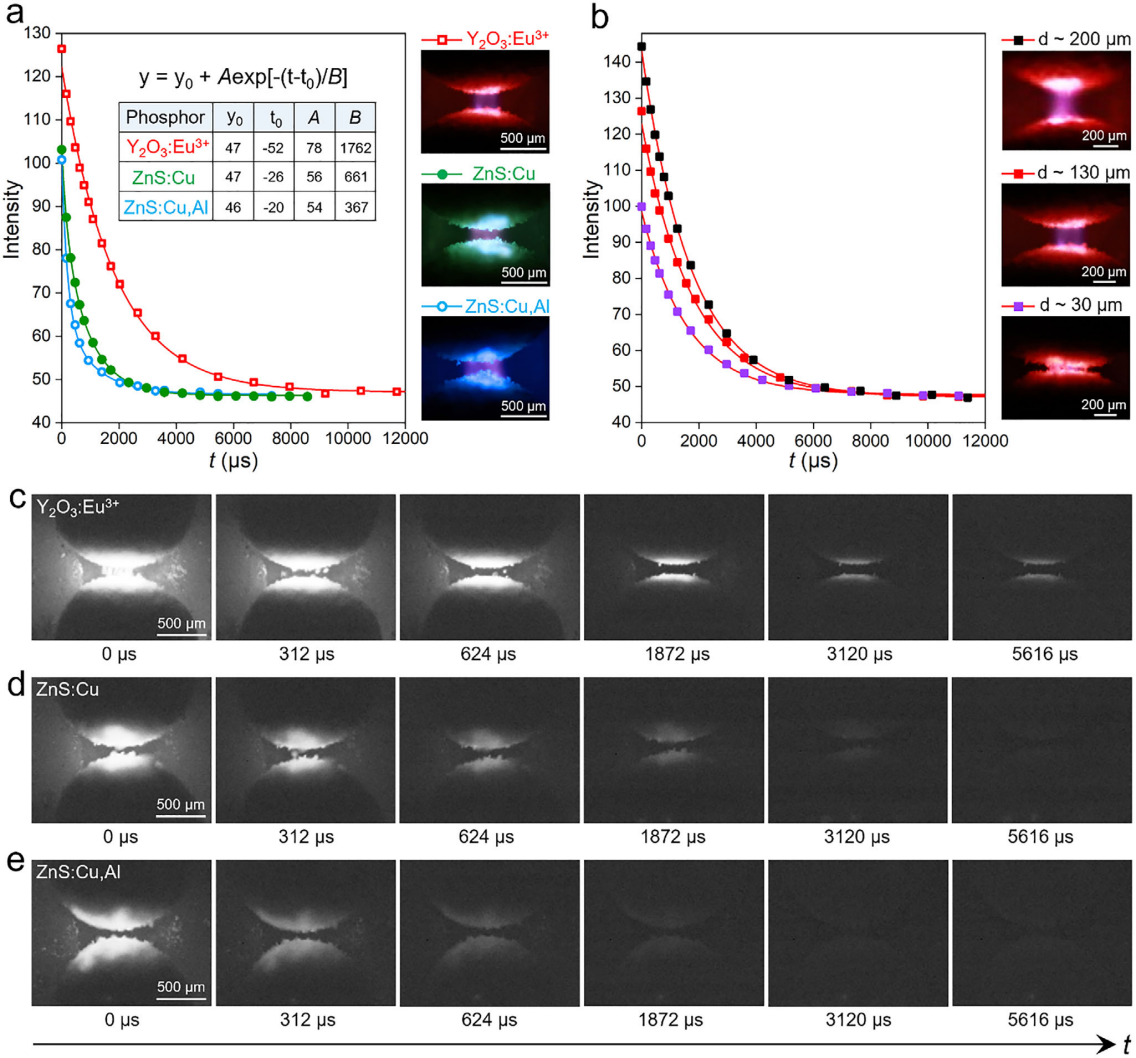
a). Area‐averaged electroluminescence intensity as a function of time for liquid metal marbles coated with Y_2_O_3_:Eu^3^⁺, ZnS:Cu, and ZnS:Cu,Al phosphor particles, during a single emission. The intensity values are calculated based on the average gray value of a fixed‐size luminescent area from the images captured with the high‐speed camera. The inset table presents the exponential fittings of the decay curves for the three phosphor types. b) Area‐averaged electroluminescence decay curves of Y_2_O_3_:Eu^3^⁺‐coated liquid metal marbles with varying inter‐marble distances (≈30, ≈130, and ≈200 µm). The optical images in Figures a and b are electroluminescent images captured using a color‐CCD camera. c–e) Time‐resolved high‐speed camera images showing the electroluminescence decay dynamics of liquid metal marbles coated with (c) Y_2_O_3_:Eu^3^⁺, (d) ZnS:Cu, and (e) ZnS:Cu,Al. The entire image in Figures c–e represents the size (area) that was used for evaluating the area‐averaged electroluminescence intensity in Figures a,b.

The temporal evolution of the discharge electroluminescence was characterized to compare the persistence and decay dynamics induced by different phosphor particles coatings. The emission triggered by a single high‐voltage pulse from individual inter‐marble gaps was recorded using a high‐speed camera at a frame rate of 64 000 frames per second. The intensity values represent the average brightness (in grayscale) over a luminescent region of the same fixed size in the high‐speed camera images. The electroluminescence emissions from different coating phosphor particles exhibit a typical exponential decay, but with markedly different afterglow durations (**Figure** **3**a). The time‐lapse high‐speed images obtained with different types of liquid metal marbles are presented in Figure 3c–e (an extended set of images can be found in Figures S8–S10, Supporting Information). The red‐light‐emitting Y_2_O_3_:Eu^3^⁺ marbles showed the longest luminescence duration, maintaining detectable brightness up to ≈8 ms. In contrast, the green‐light‐emitting ZnS:Cu marbles and the blue‐light‐emitting ZnS:Cu,Al marbles displayed a progressively shortened afterglow, which faded into the background level within ≈3 ms. These differences align with the intrinsic afterglow properties of each phosphor. These trends are consistent with the known decay characteristics reported in the literature: Y_2_O_3_:Eu^3^⁺ phosphors exhibit long afterglow durations on the order of several milliseconds due to the deep trap states that store excitation energy and the resulting slow release,^[^ ^27^ ^]^ while the ZnS:Cu and ZnS:Cu,Al phosphors display significantly shorter decay times as their excited carriers recombine more rapidly with fewer trap‐assisted delays, resulting in fast emission and rapid return to the ground state.^[^ ^28^ ^]^

The influence of inter‐marble distance on the electroluminescent effect was analyzed by investigating the decay dynamics at three gap distances (namely, ≈30, ≈130, and ≈200 µm), using the Y_2_O_3_:Eu^3^⁺‐coated liquid metal marbles and a fixed triggering voltage (Figure 3b). The area‐averaged electroluminescence intensity curves reveal that both the onset and time‐dependent (integrated) emission intensities increased at larger gap distances. This can be attributed to the increased illuminating area at larger gap distances. It also suggests that when a sufficiently high voltage was applied (as in the current case), the peak intensity can be largely maintained when the inter‐marble distance increases within the distance range investigated, which offers a way for enhancing the electroluminescent effect.

### 2.4 Tuning Particle Mixing Ratio for Electroluminescent Additive Color Mixing

**Figure 4:**
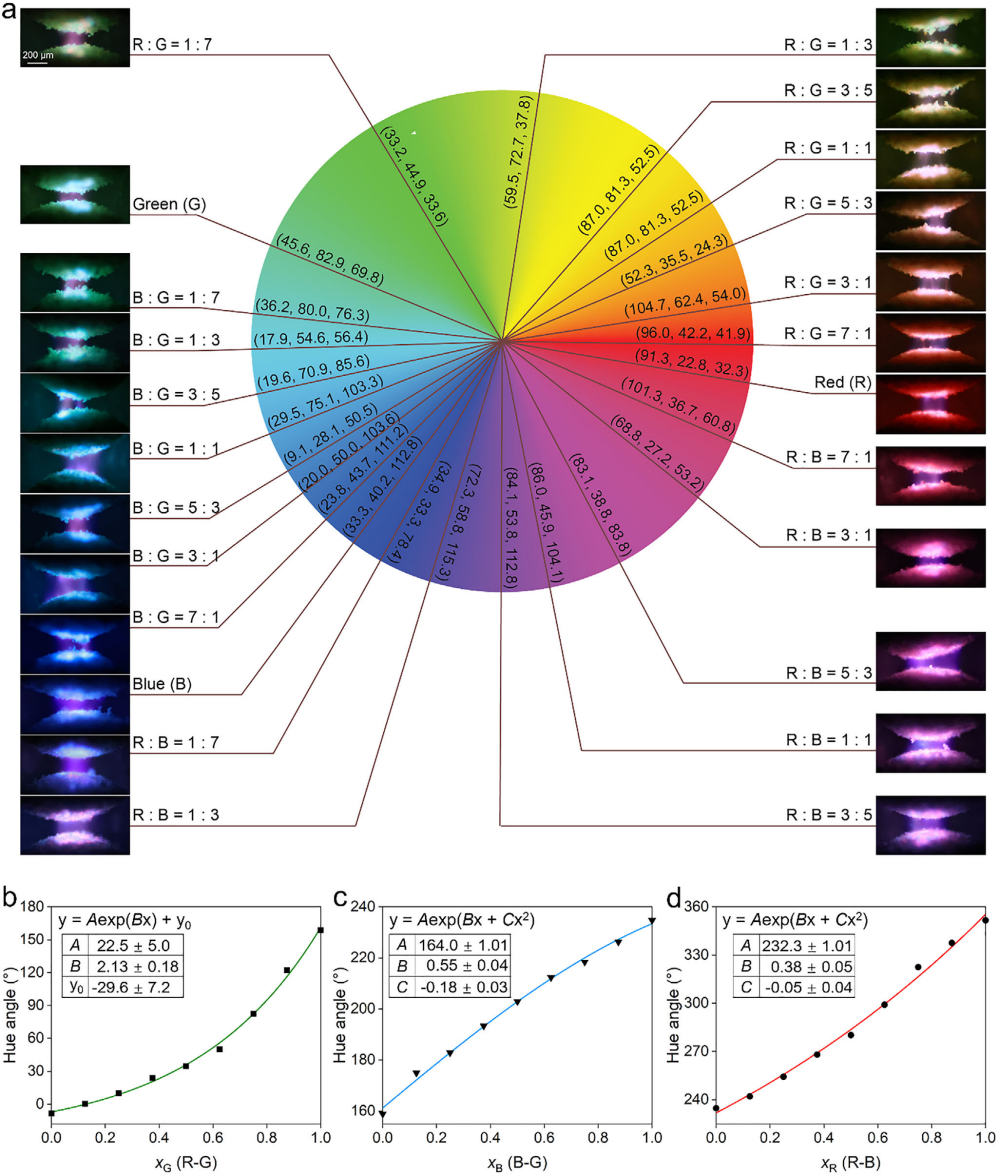
a). Additive colors emitted by liquid metal marbles coated with phosphor particles mixed at varied ratios on a RGB color wheel. An optical image of each combination is given together with the corresponding coating phosphor particle types, their mixing ratios, and the RGB values of emitted color. b–d) Hue angle variations as function of mixing ratios between the R‐G (b), B‐G (c), and R‐B (d) phosphor combinations. The scatters are experimental results, and the lines are exponential fitting curves with the fitting parameters listed in each figure.

As has been shown in Figure 1f–h, mixing different types of phosphor particles effectively tunes the electroluminescent color emitted by the liquid metal marbles. To further demonstrate the color tunability with our method, we characterize the electroluminescent color of liquid metal marbles coated with two types of phosphor particles at varied ratios. As shown in **Figure** **4**, a series of 24 representative formulations, including the primary‐color‐emitting Y_2_O_3_:Eu^3^⁺ (red), ZnS:Cu (green), and ZnS:Cu,Al (blue) phosphor particles and their binary mixtures, were systematically inspected as the marble coating. The electroluminescent color obtained from each coating combination was quantified in the RGB scale (Experimental Section). The averaged RGB values were then converted to Hue–Saturation–Value (HSV) color space to extract the hue component, allowing the assignment of each color on a standard color wheel.

The liquid metal marbles with a single type of phosphor particle coating generated emissions with well‐defined hue positions that are close to those of the three primary colors on the color wheel. Binary phosphor particle combinations gave rise to progressive chromatic transitions within the range defined by the primary colors of the constituent phosphors. For instance, increasing the ratio of green in R: G combinations caused the hue angle to shift gradually from red to orange (R: G = 3: 1), yellow (R: G = 3: 5), and lime green (R: G = 1: 3). Similar color transitions as a result of mixing ratio tuning were also observed for the G: B and R: B groups. It can be expected from these results that more colors across the color spectra can be realized by fine tuning the particle mixing ratios.

A closer look at the emission zone between marbles with a R: B = 1: 3 mixing ratio reveals that, individually, the Y_2_O_3_:Eu^3^⁺ and ZnS:Cu,Al particles emit their characteristic red and blue color, respectively (Figure S11, Supporting Information). The two primary colors blend at the microscopic scale defined by the particle sizes, and due to the spatial averaging at such small scales, the emission is precepted as a purple additive color. The color fusion principle of our liquid metal marbles is the same as that of LED or commercial plasma system displays.^[^ ^12^, ^13^, ^29^ ^]^ This means that fined tuned and well blended additive color mixing is achievable by simply mixing the phosphor particles at adjustable ratios using our method.

Despite the randomness in phosphor placement and discharge spark position, the liquid metal marble platform produces macroscopically uniform colors through the optical averaging effect.^[^ ^14^, ^30^ ^]^ These findings show the feasibility of using the liquid metal marble platform for tunable, additive color mixing and display while requiring no microscopic control of the color‐emitting components. In addition, the emitting colors reflect the types and mixing ratios of the particles coating on the liquid metal marbles. Provided that the types of electroluminescent particles are known, it is possible to obtain the types and mixing ratios (quasi‐quantitatively) of the particles.

Noted that the three types of phosphor particles used in this study have comparable microscopic sizes and morphologies (Figure 2a–c). When more than one type of phosphor particles was used, the mixture was thoroughly homogenized using a vortex mixer prior to particle coating. Following these procedures, the liquid metal marbles showed good consistency in terms of particle coverage and luminescent colors (indicating consistent particle mixing ratio) across different batches. The hue angle change as a function of mixing ratio for different binary particle combinations can be fitted using exponential models, indicating smooth and controllable color tuning with our additive color mixing strategy (Figure 4b–d).

### 2.5 Reconfigurable Multi‐color Display Demonstrations

**Figure 5:**
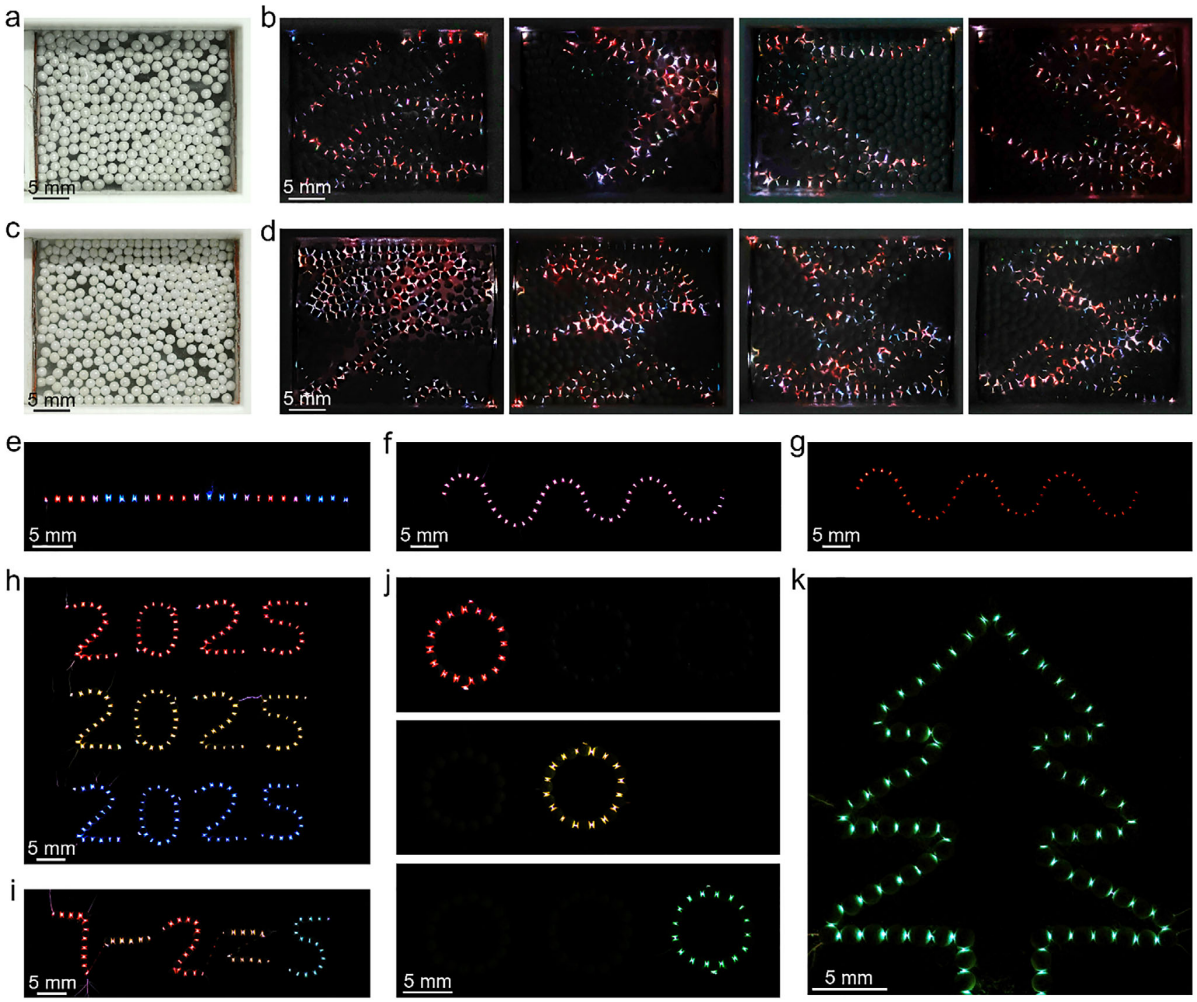
a,b) Visualization of colored discharge paths formed in a randomly packed liquid metal marble assembly. c,d) Visualization of discharge paths in a similarly packed assembly composed of smaller liquid metal marbles (1.1 µL), leading to denser electroluminescent emission points and enhanced spatial resolution compared to the larger marbles (1.6 µL) shown in a,b. e) Alternating color emission from a linear marble chain. f,g) Emission color switching from a sinusoidal chain during a single emission event. h–k) Multi‐color display with liquid metal marble arranged into different patterns: alphanumeric symbols of different color emission (h), a symbolic math equation (i), circular marble arrays mimicking traffic light signals (j), a “Christmas tree” shaped pattern (k).

To demonstrate the versatility and programmability of liquid metal marble‐based multi‐color display platforms, liquid metal marbles with different emitting colors were assembled into different layouts and patterns. A randomly packed liquid metal marble assembly was configured to demonstrate multi‐color emitting from its dynamically evolving discharge paths (**Figure** **5**a,b; Video S1, Supporting Information). It visualizes the dynamic nature of the electric discharge, resembling the interactions in granular systems.^[^ ^9^, ^31^ ^]^ Further miniaturization of the liquid metal marbles (from 1.6 to 1.1 µL) was found to reduce inter‐pixel spacing (more electroluminescent pixel along the same distance) and increase emission density, thereby enhancing the spatial resolution (Figure 5c,d). We note that 1.1 µL is the minimum droplet size that can be obtained using our droplet generation method with the available syringe pump method in our work. We used the same marble fabrication procedure across this work to ensure consistency in this study. However, we expect that the implementation of other approaches based on, for example, microfluidics or ultrasonication, could achieve further marble miniaturization for improving display resolution.

A linear chain made of alternating red‐ and blue‐light‐emitting marbles in Figure 5e (Video S2, Supporting Information) shows the feasibility of controllable color display. In the chain of a sinusoidal‐shape pattern made of marbles coated with red‐blue phosphor particles (mixing ratio 5:3), a pink emission is observed during the earlier stage of emission, which quickly turns red (Figure 5f,g; Video S3, Supporting Information). This color switching behavior can be understood by considering the faster decay of the blue light produced by the ZnS:Cu,Al particles compared to the red light by the Y_2_O_3_:Eu^3^⁺ particles. This temporal decay mismatch resulted in a visible color shift from pink to red during individual emitting events, highlighting the capability of the liquid metal marble display platform for encoding temporal information. Multi‐color alphanumeric symbols and various geometries were further fabricated to display complex patterns (Figure 5h–k; more examples in Figures S12 and S13, Supporting Information). Their vivid, tunable colors, and reconfigurable geometries indicate that the liquid metal marble platform could be used as an easy‐to‐fabricate electroluminescent multi‐color display system. It is also suggested that the liquid metal marble platform, presented in this work, could offer strong potential for future integration with automated manipulation systems.

## 3 Conclusion

In this work, we demonstrated a reconfigurable multi‐color pixelated display platform based on the electric discharge of phosphor‐coated liquid metal marbles. The native gallium oxide shell on EGaIn droplets played a crucial role in enabling conformal and stable phosphor particles adhesion, while a proposed thermal annealing step further enhanced the durability under repeated discharge cycles. The platform allowed additive RGB color mixing through the direct physical mixing of primary‐color‐emitting phosphor particles, enabling full‐color outputs. Given the particle level uniform mixing and optical averaging during perception, such a particle‐based additive color mixing strategy could produce colors spanning the entire visible spectrum. In addition, the freeform configurations of the liquid metal marbles further allowed a wide range of reconfigurable discharge patterns with programmable colors to be realized. Our liquid metal‐based plasma platform offers a compelling alternative to conventional display technologies, with potential applications in interactive displays, artistic installations, and electrooptic sensing and detection. It enables fine tuning the electroluminescence coatings of liquid metal marbles for pixelated multicolor displays, realizing reconfigurable light‐emitting patterns without the need for physical wiring between pixels. It also allows the design and fabrication of multicolor patterns and displays on demand.

## 4 Experimental Section

### Materials and Methods for Marble Fabrication

Liquid metal marbles with an average diameter of ≈1.2 mm were fabricated using EGaIn, prepared by mixing and melting gallium (99.999%) and indium (99.99%) that were purchased from Rotometals. EGaIn droplets with controlled volume were extruded using a syringe pump (Fusion 200, Chemyx Inc.) and subsequently rolled over a phosphor powder bed to achieve uniform surface coating. Three types of commercial phosphor powders, namely, Y_2_O_3_:Eu^3^⁺ (HU9196, 3–4 µm), ZnS:Cu (D512S, ≈9 µm), and ZnS:Cu,Al (D417S, ≈9 µm), were utilized to generate the red, green, and blue primary colors, respectively. Phosphor powders were supplied by Shanghai Keyan Phosphor Technology Co., Ltd. Phosphor particle mixing was achieved by mixing selected phosphor types at specific ratios using a vortex mixer (CORNING). A thermal annealing step at 200 °C for 30 min (in ambient air) was introduced to improve the stability of the marbles.

### Imaging Electric Discharge Events

Electric discharge was triggered using a compact Tesla coil (10MINI, Wuhan Stark Technology Co., Ltd). The output voltage and output frequency were 18 kV (estimated based on the maximum discharge path length) and 25 Hz, respectively. To capture the electric discharge events, a Canon EOS R8 digital camera was mounted onto an optical microscope (BX53M, OLYMPUS) via a C‐mount camera adapter and a lens converter. Images were captured using a 10× objective under darkroom conditions, with the exposure set to 1/30 s and ISO set to 20000. The images were analyzed using the ImageJ Software package to extract RGB values and to compute hues. For image processing, pixels with grayscale brightness values ≥ 68 were selected to capture the discharge region and compute average RGB values. The RGB values were then converted into HSV space to extract the hue component using the following standard color space transformation formulas.

(1) $$\theta =cos^{-1}\left[\frac{1}{2}\cdot \frac{\left(R-G\right)+\left(R-B\right)}{\sqrt{{\left(R-G\right)}^{2}+\left(R-B\right)\left(G-B\right)}}\right]$$

(2) $$Hue=\left\{\begin{array}{c} \theta ,\;if\;B\leq G\\ 360^{\circ }-\theta ,\;if\;B>G \end{array}\right.$$

where *θ* is the anglar position of a given color on the color wheel, *R*, *G*, and *B* represent the three primary color values. To characterize the time‐resolved dynamics of the electric discharge events, high‐speed imaging was performed using a monochrome CCD camera (VEO610L, PHANTOM) at 64 000 frames per second with an exposure time of 2 µs. The brightness was quantified using ImageJ based on the grayscale of the images.

### Multi‐Color Display Pattern Demonstration

Liquid metal marbles coated with different phosphor compositions were configured on a flat polylactic acid (PLA) substrate to form programable patterns. Exemplar patterns include alphanumeric chains such as “2025” and “NSW” and information‐containing shapes and symbols. Color images were captured using a Canon EOS R8 camera with a Canon RF 35 mm F/1.8 IS Macro STM lens (1/30 s exposure, ISO 2500, and f/3.0 aperture under darkroom conditions).

## Conflict of Interest

The authors declare no conflict of interest.

## Supporting information



Supporting Information

Supplemental Video 1

Supplemental Video 2

Supplemental Video 3

## Data Availability

The data that support the findings of this study are available in the supplementary material of this article.
