# Supplementary material for: Electroluminescent Liquid Metal Marbles for Reconfigurable Multi‐Color Display
Source: Adv Sci (Weinh). 2025 Aug 28;12(43):e12263. doi: 10.1002/advs.202512263 (PMC12631868; doi:10.1002/advs.202512263)
Supplement: Supplementary file 1 — Supporting Information [file ADVS-12-e12263-s001.docx]

Supporting Information

**Electroluminescent Liquid Metal Marbles for Reconfigurable Multi-Color Display**

*Ruohan Yu, Yuan Chi, Richard Fuchs, Shih‐Hao Chiu, Yuanzhu Mao, Shuhua Peng, Priyank Kumar, Kourosh Kalantar-Zadeh*, Jianbo Tang**


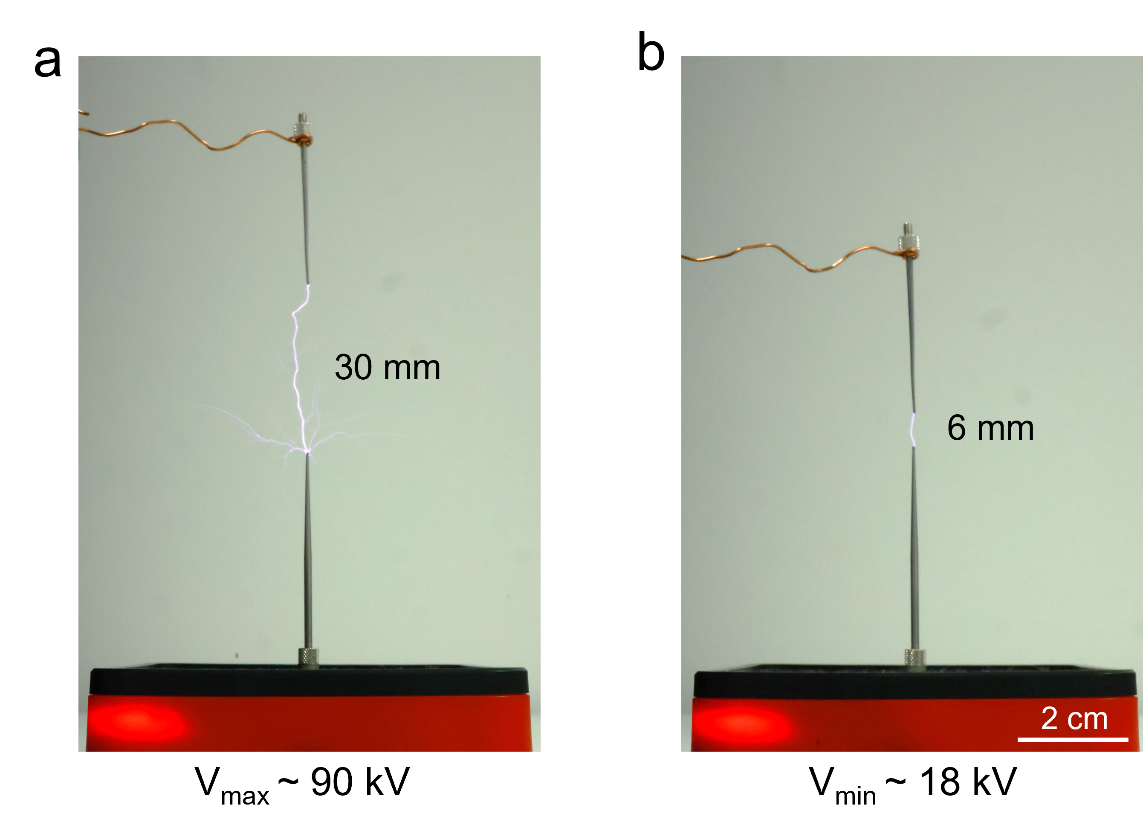


**Figure S1.** A compact Tesla coil served as the voltage source in this study, generating a high-pulse electric field with manually adjustable output voltage and frequency. The optical images show air breakdown between two needle electrodes under ambient conditions. The maximum discharge arc length reached ~30 mm (a), corresponding to an estimated voltage of ~90 kV based on Paschen’s law. The minimum arc length observed was ~6 mm (b), equivalent to ~18 kV. All discharge events in this work were conducted under the minimum voltage setting to ensure stable excitation while avoiding damage to the phosphor-coated marbles.


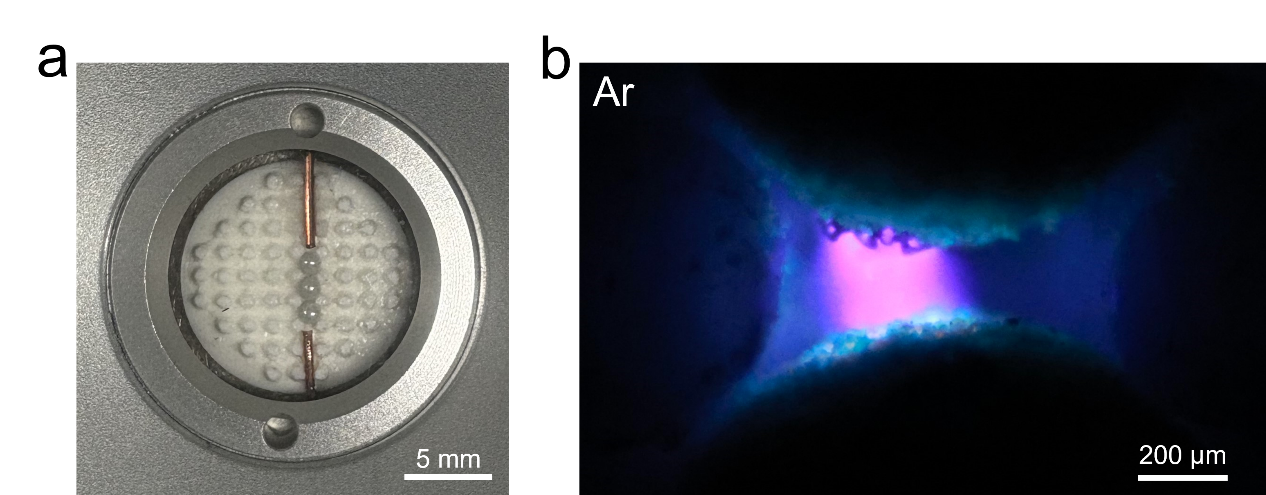


**Figure S2.** a) Marble array loaded into a gas-tight Linkam stage. b) Plasma emission observed under an argon atmosphere, exhibiting intense and vibrant purple light.


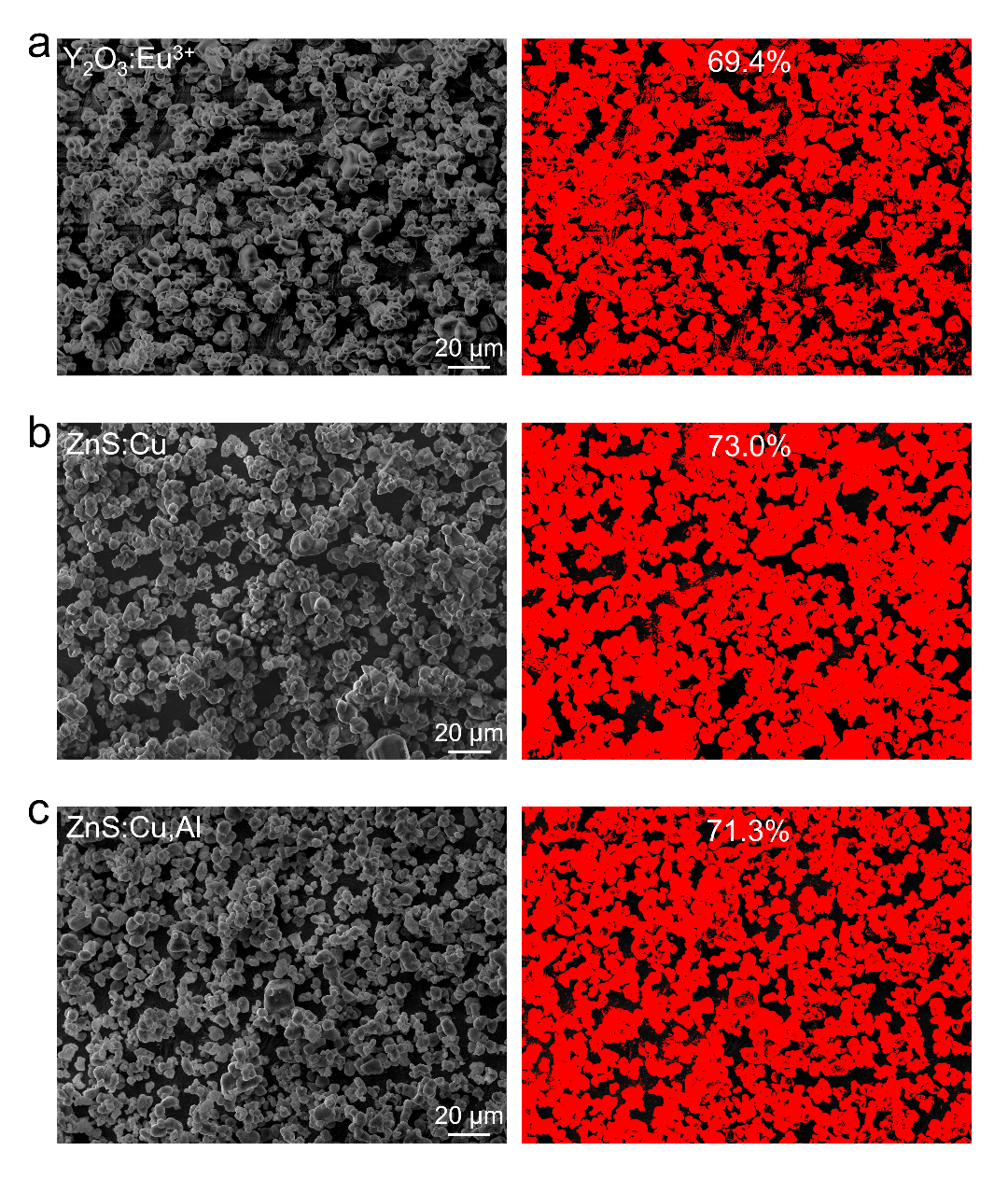


**Figure S3.** Particle surface coverage analysis of phosphor-coated liquid metal marbles for different phosphor types. a-c) Representative SEM images (left) and corresponding surface maps (right) for marbles coated with (a) Y₂O₃:Eu³⁺, (b) ZnS:Cu, and (c) ZnS:Cu,Al particles. The red regions indicate phosphor-covered areas identified using ImageJ. The calculated surface coverage for each sample is 69.4%, 73.0%, and 71.3%, respectively, confirming consistently high coverage across all phosphor types.


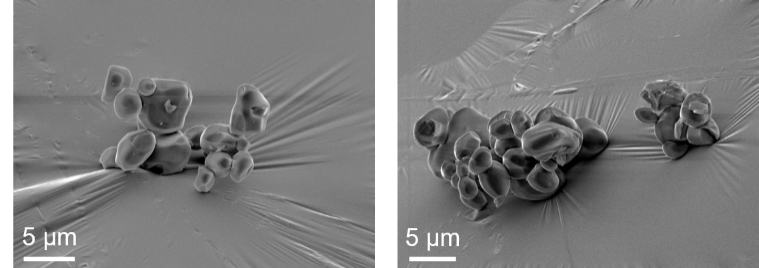


**Figure S4.** SEM images of phosphor particles embedded on the oxidized surface of a liquid metal marble. The native gallium oxide layer acts as a stabilizing scaffold, enabling the tight anchoring of individual phosphor particles. It ensures robust particle attachment under external excitation and is critical to maintaining functional electroluminescence during repeated discharge operations.


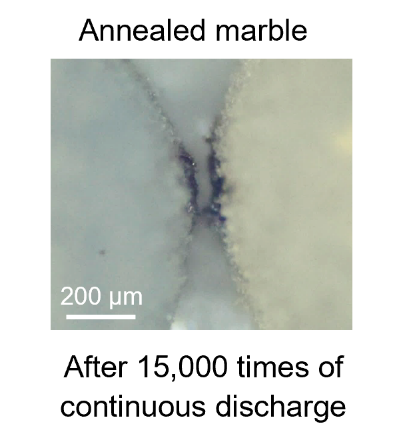


**Figure S5.** Optical image of adjacent annealed liquid metal marbles after 15,000 discharge cycles.


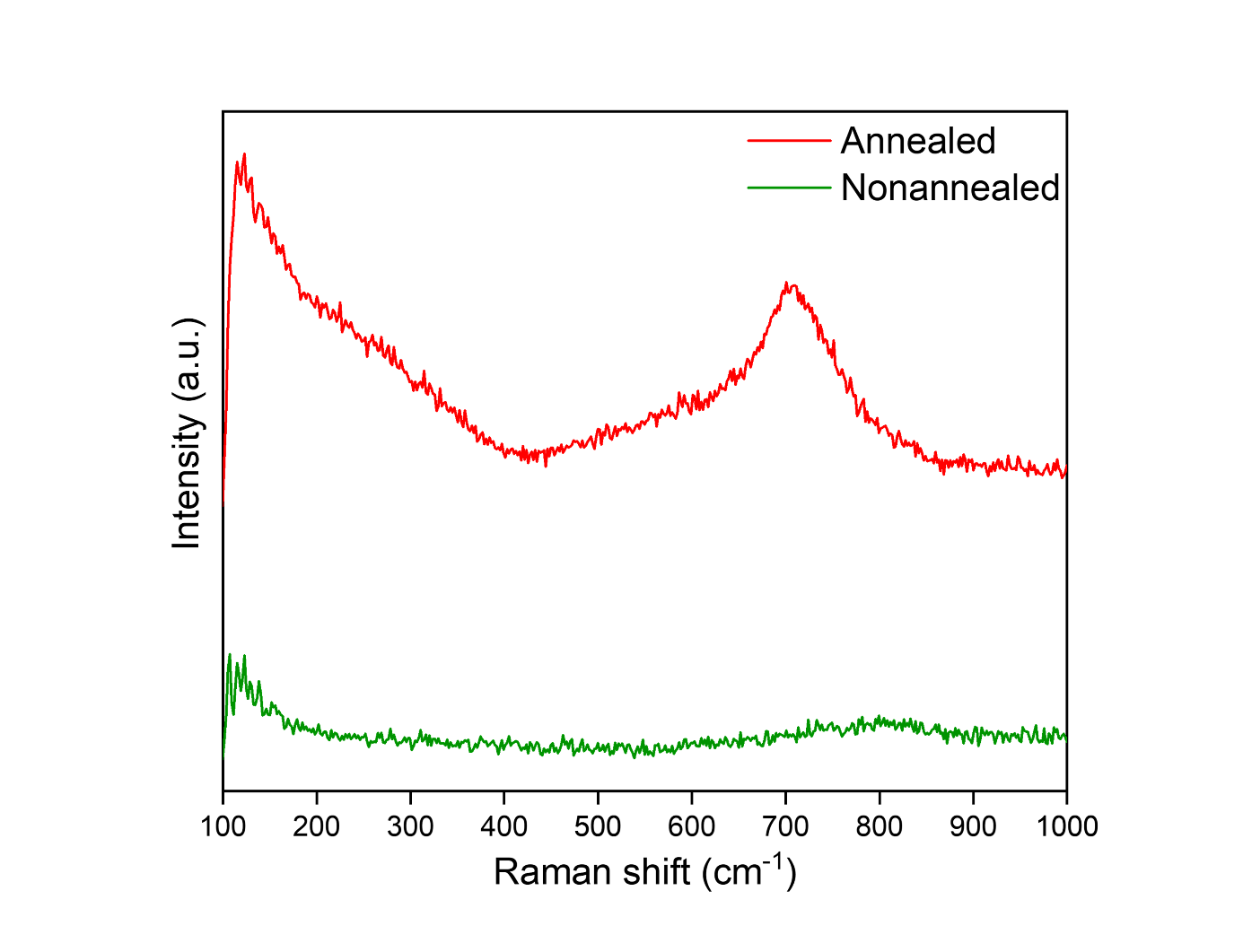


**Figure S6.** Raman spectra of EGaIn droplets before and after annealing.


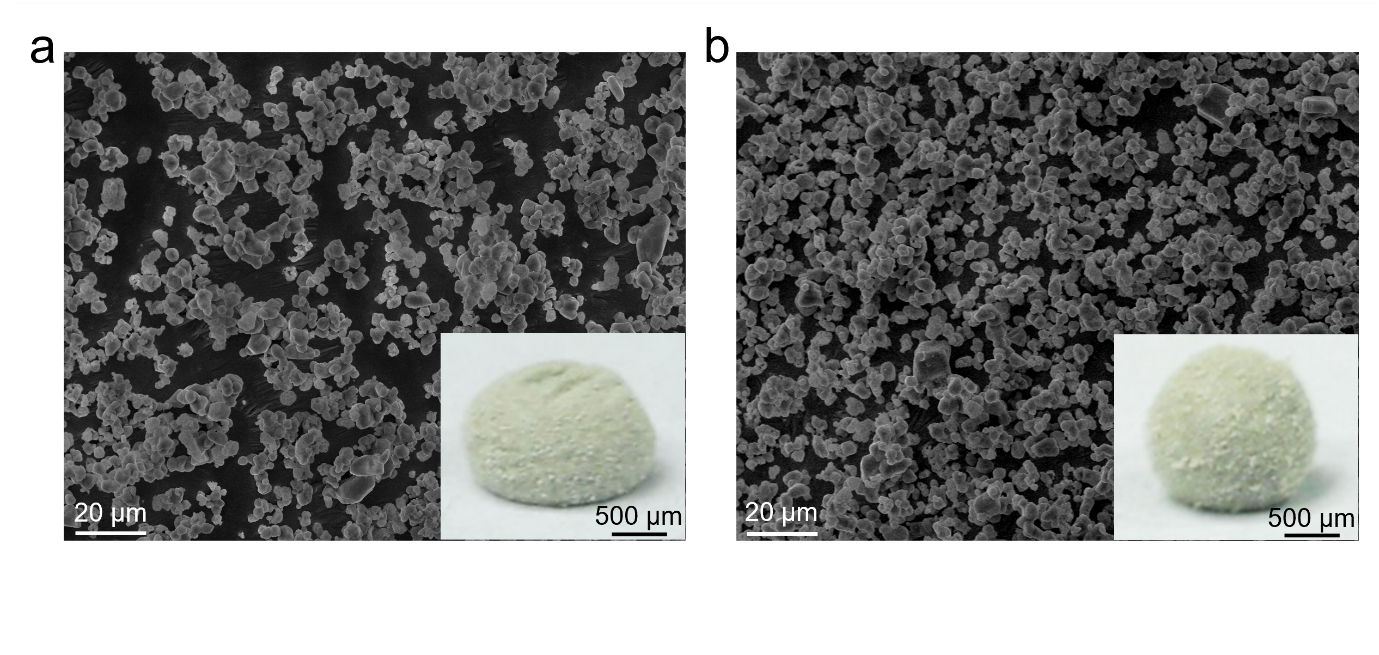


**Figure S7.** SEM images of liquid metal marbles subjected to comparable shaking treatment for annealed (a) and not annealed (b) samples. The non-annealed marbles show significant particles detachment and structural collapse, while the annealed marbles maintain high particles coverage and spherical shapes. Insets show the images of marbles, annealed and non-annealed after mechanical treatment.


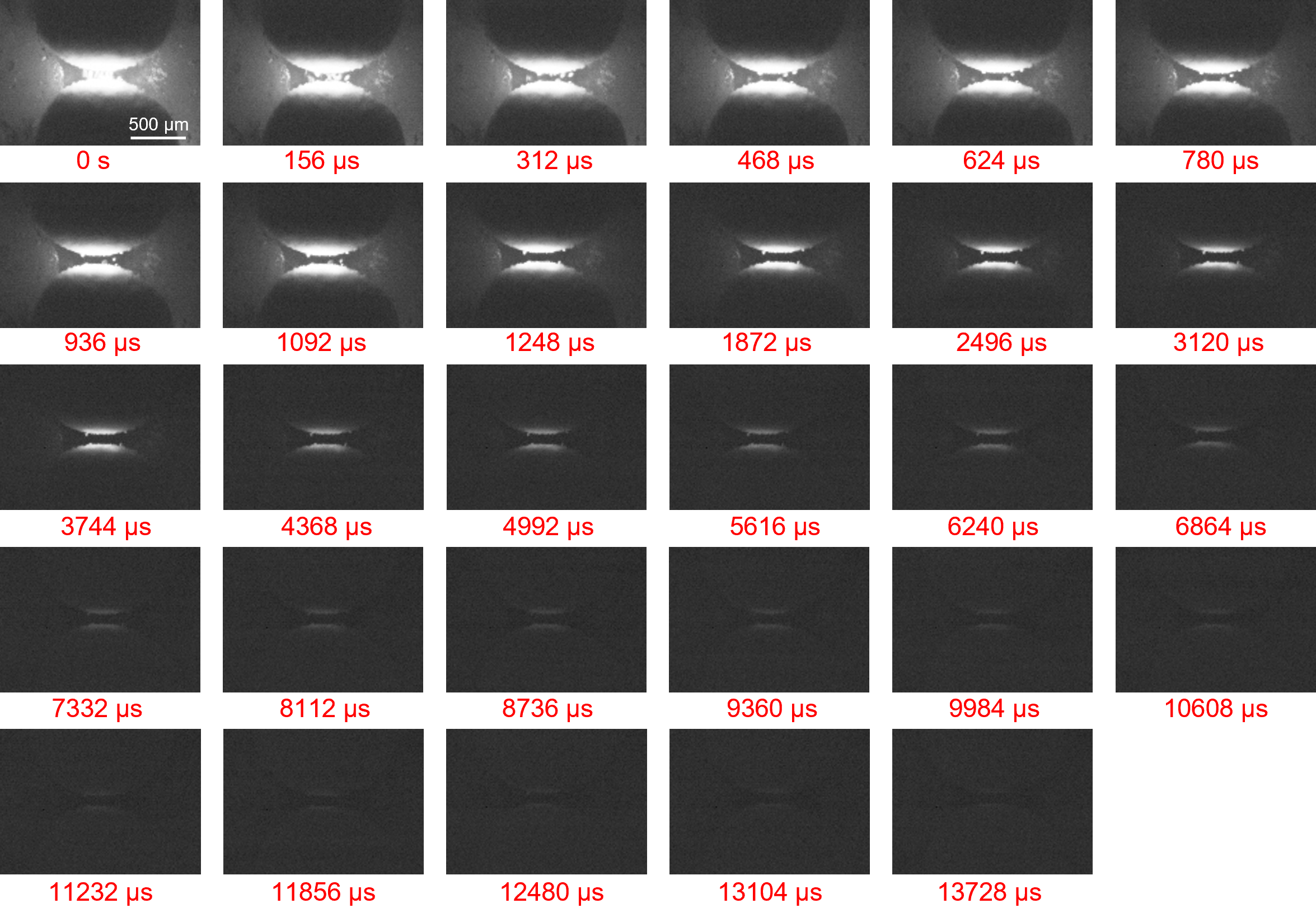


**Figure S8.** Extended high-speed camera images showing the electroluminescence decay of liquid metal marbles coated with Y₂O₃:Eu³⁺ particles. The frame rate was 64,000 frames per second.


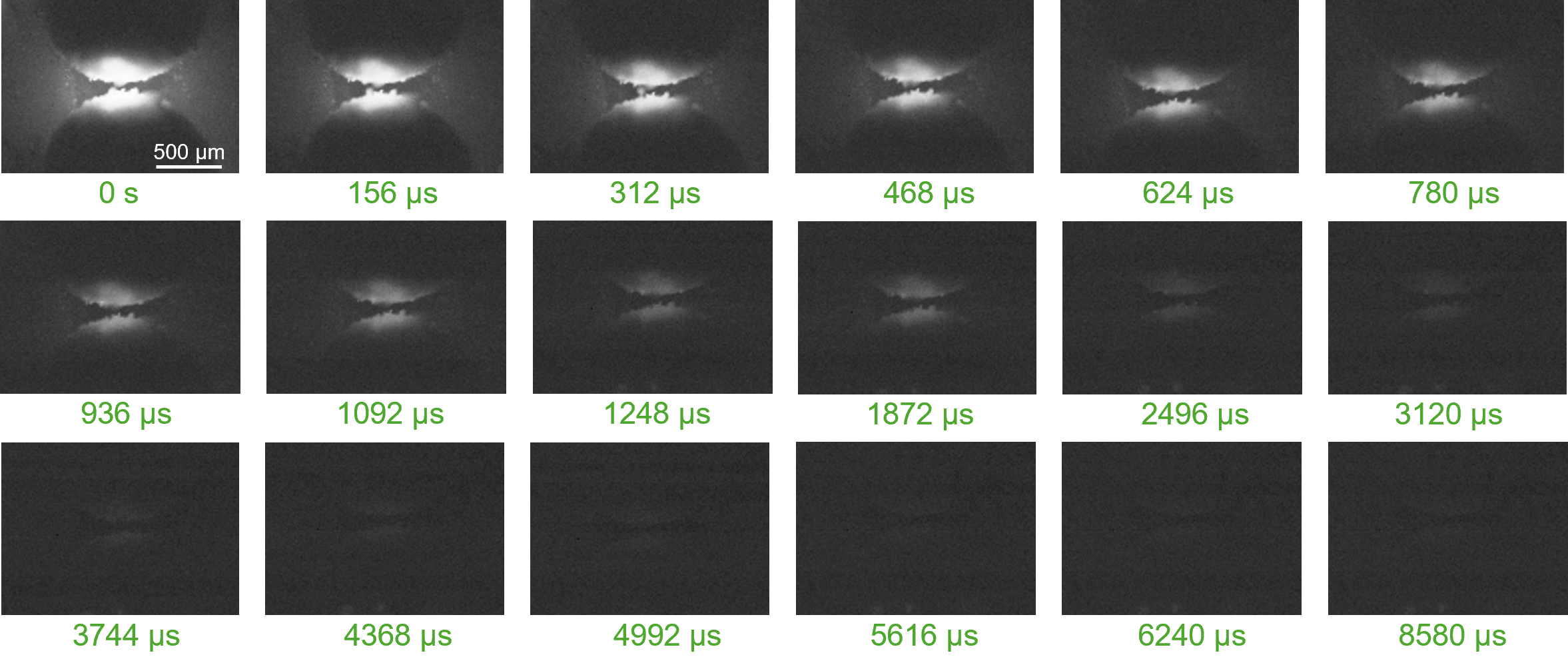


**Figure S9.** Extended high-speed camera images showing the electroluminescence decay of liquid metal marbles coated with ZnS:Cu particles. The frame rate is 64,000 frames per second.


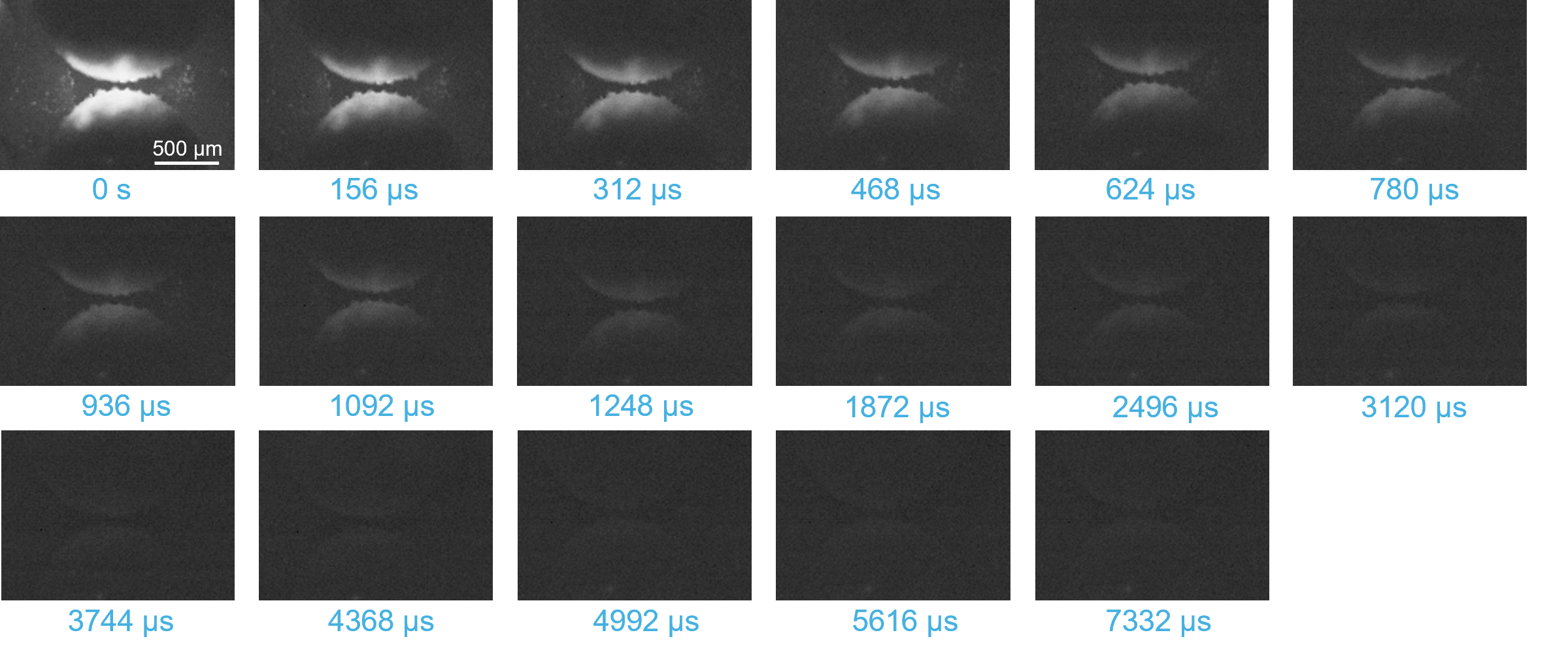


**Figure S10.** Extended high-speed camera images showing the electroluminescence decay of liquid metal marbles coated with ZnS:Cu,Al particles. The frame rate is 64,000 frames per second.


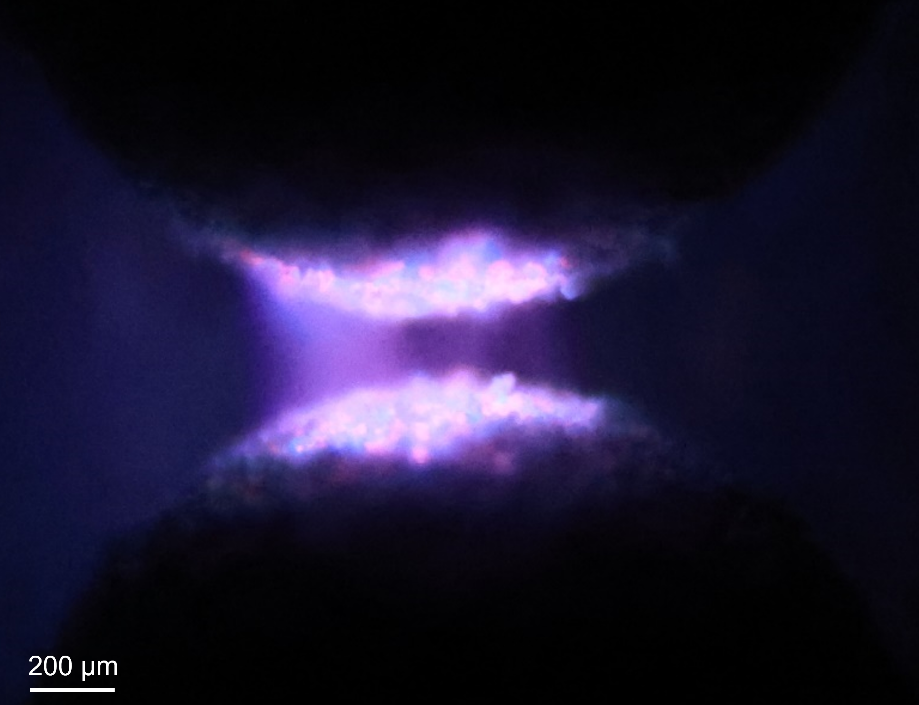


**Figure S11.** Magnified emission zone between two adjacent liquid metal marbles (R : B = 1 : 3), showing discharge-induced electroluminescence at the inter-marble gap. Red- and blue-emitting phosphor particles are visibly distributed across the marble surfaces and simultaneously excited during discharge. Despite the distinct emission origins, the overlapping luminescence is perceived as a uniform purple hue, illustrating the principle of additive color mixing.


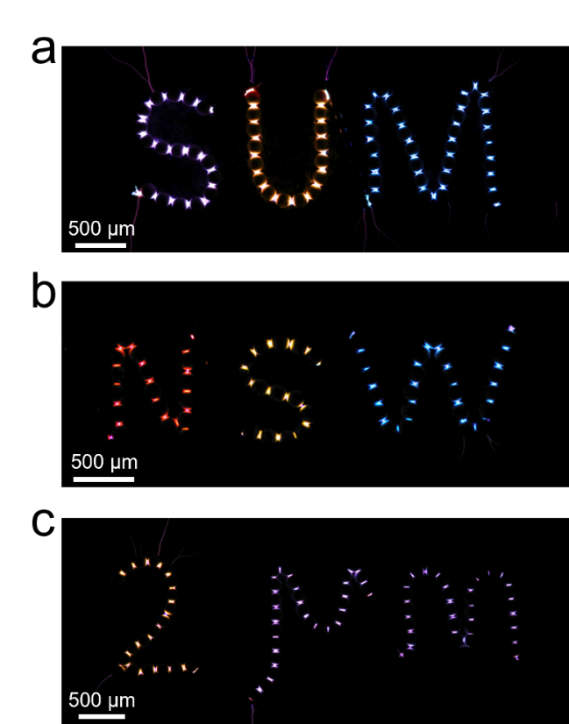


**Figure S12.** Text-based patterns displayed using liquid metal marbles. Examples such as “SUM”, “NSW”, and “2 μm” demonstrate the system’s capacity for flexible and customizable alphanumeric display. Different color combinations (e.g., red + green = yellow; red + blue = magenta) were used to enhance visual contrast.


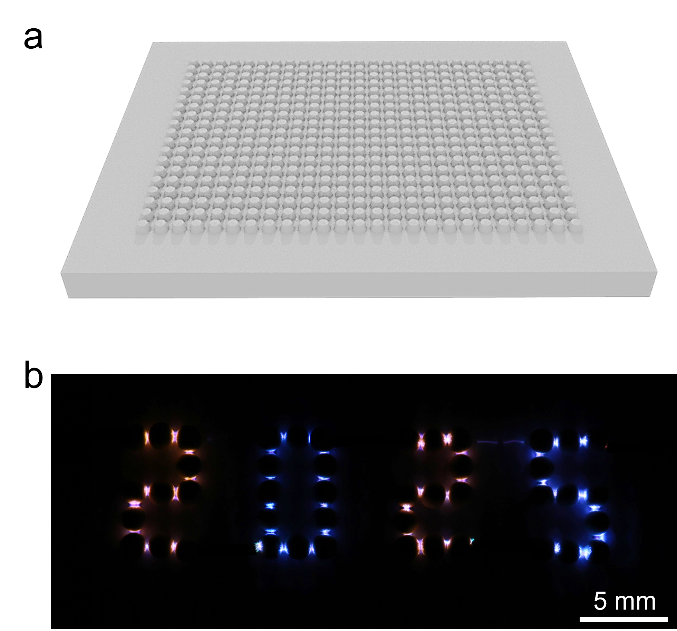


**Figure S13.** a) Schematic of a 3D-printed holder designed to spatially confine liquid metal marbles into a regular pixel array. b) Optical emission image of “2025” formed using the 3D-printed holder, demonstrating pixel-level alignment and reproducibility under high-voltage excitation.
